# Hybrid noise reduction-based data-driven modeling of relative humidity in Khulna, Bangladesh

**DOI:** 10.1016/j.heliyon.2024.e36290

**Published:** 2024-08-14

**Authors:** Shuvendu Pal Shuvo, Joarder MdAshikuzzaman, Shirshendu Pal Shibazee, Goutam Paul, Pritam Banerjee, Kazi Mashfiq Fahmid, Ashiqur Rahman

**Affiliations:** a Department of Civil Engineering, Khulna University of Engineering & Technology, Khulna, Bangladesh; b Department of Electrical and Electronic Engineering, Bangladesh University of Engineering and Technology, Dhaka, Bangladesh; c Department of Pharmacy, Bangabandhu Sheikh Mujibur Rahman Science and Technology University, Gopalganj, Bangladesh

**Keywords:** Relative humidity, Empirical mode decomposition (EMD) and support vector machine (SVM)

## Abstract

In this study, a hybrid Machine Learning (ML) approach is proposed for Relative Humidity (RH) prediction with a combination of Empirical Mode Decomposition (EMD) to improve the prediction accuracy over the traditional prediction technique using a Machine Learning (ML) algorithm called Support Vector Machine (SVM). The main objective of proposing this hybrid technique is to deal with the extremely nonlinear and noisy humidity pattern in Khulna, Bangladesh, which is experiencing rapid urbanization and environmental change. To develop the model, data on temperature, relative humidity, rainfall, and wind speed were collected from the Bangladesh Meteorological Department (BMD), and the data was divided into three phases: 70 % of the historical dataset as training data for the model, 15 % of the data set as the validation phase, and the remaining 15 % of the data set as the test phase of the model. Employing the Particle Swarm Optimization (PSO) algorithm, the SVM model determines its best hypermeters within this research. In the present research, performance analysis is carried out utilizing the Mean Square Error (MSE), Root Mean Square Error (RMSE), Mean Absolute Error (MAE), Mean Absolute Percentage Error (MAPE) and Coefficient of Determination (R2). Results show that the increase in R2 values resulting from the EMD-based approach is significant: 21.05 % in H1(Traditional model), 19.48 % in H2 (Traditional model), 76.92 % in H3 (Traditional model), 55.93 % in H4 (Traditional model), and 64.29 % in H5 (Traditional model) and H6 (Traditional model). The analytical results show that the proposed EMD-based technique efficiently filters and processes noisy, highly nonlinear humidity data during prediction in the Khulna region. It is recommended that this technique could be applied to other geological areas.

## 1 Introduction

Relative humidity prediction is vital in a variety of fields, having a significant impact on weather forecasting, agriculture, health, and providing basic comfort levels in people's daily life. Accurate humidity forecasts will help meteorologists to forecast weather condition more precisely and provide better preparation against extreme weather such as storms, floods, and heatwaves [1]. Humidity information helps farmers in agriculture to know the irrigation schedules for their crops, avoiding crop diseases, and enhancing yield quality. It can help minimize respiratory problems, reduce airborne diseases, and generally improve indoor air quality for health if the humidity is well controlled. Also, excessive humidity aggravates heat stress. Accurate predictions are required, as public health recommendations are especially important during heatwaves [2]. These also benefit applications in technology; an example is that humidity sensors have a huge stake in HVAC systems to create comfortable indoor environments and industries where the process requires an ideal range of relative humidity [3]. In the field of aviation, correct humidity data is essential for safe flights and fuel efficiency. It also impacts the life and integrity of buildings and infrastructure because it influences durability and performance based on the characteristics of materials. On a larger environmental scale, trends in humidity serve researchers undertaking climate studies and artfully modelling future scenarios for the effects of climatic changes [4]. Joining the modern advancements in data collection and predictive analytics with illuminating humidity forecasts in smart city initiatives serves as an integration in the improvement of urban living standards through effective management of air quality and energy consumption [5]. Basically, air humidity prediction is a multi-dimensional tool of great importance for facilitating decision-making in different applications, which further enhances basic safety, efficiency, and sustainability [4,6].

Nowadays, due to the rapid changes in climate, humidity forecasting has been of great concern to researchers. But it is very difficult to analyze the intricate humidity pattern because of its high nonlinearity, existing noise, and dependency on other factors. For predicting humidity, researchers have used two famous approaches, such as the time series model and the machine learning model. The most commonly time series model used among them are SARIMA [[7], [8], [9], [10]], ARIMA [11], and ARMA [12] models. Even with their wide applications, time series models tend to have higher error rates in humidity predictions because most of the time, humidity data shows non-linearity, while time series models are based on linearity assumptions. Humidity is influenced by a variety of external factors, including geography, climate, and environmental conditions. Furthermore, ARMA, ARIMA and SARIMA models exclude external variables, which could reduce their ability to predict humidity. In this regard, time series models can become ineffective predictors of extreme events. The machine learning models, on the other hand, improve the prediction accuracy of these significantly by effectively capturing complex nonlinear patterns that traditional time series models might not handle [13]. The ML models are able to capture the non-linear pattern by non-linear relationships [14]. Artificial Neural Networks [[15], [16], [17]], Support Vector Machines [18,19], Long Short-Term Memory Networks [20,21], Adaptive Neuro Fuzzy Inference System (ANFIS) [22] and Decision Trees [23] are common ML models used in humidity prediction. These have shown better results by virtue of their ability to model complex dependencies and changes in the humidity data. Also, integration of ML models has taken place with the classical time series approaches, arriving at hybrid models in such a way that these shall use the power of both [24]. These combined models increase prediction accuracy to a large extent [25]. It is on the basis of the aggregate performance of multiple models that ensemble methods barely manage to enhance predictive performance, subduing or lessening the weaknesses of the individual models and preserving their strengths [[26], [27], [28], [29], [30]].

Weather data are usually noisy due to measurement errors and the inherently nonlinear environmental factors, which complicate the extracting of intricate patterns. Hence, noise can considerably bring down the performance of machine learning models. It can reduce the performance of the ML models. In this study, this paper proposes an Empirical Mode Decomposition (EMD) - based denoising approach to mitigate such limitations by coupling with the SVM model. EMD is well-suited for breaking down such data into a set of intrinsic mode functions (IMFs) that capture the various scales and patterns contained in the data [31,32]. The novelty lies in the fact that this research uses an Empirical Mode Decomposition method to gain an elaborated analysis on the prediction of humidity. EMD is able to enhance the ability of the SVM model to capture more intricate patterns, thus improving the overall accuracy and depth of analysis. Empirical Mode Decomposition has been widely applied to several fields, including rainfall prediction [31], speed of wind [33], monthly streamflow prediction [34],ground water level prediction [35], pH prediction of water treatment plant [36] etc. This makes the use of EMD very important in improving predictive models and understanding phenomena.

To compare the model's performance, three distinct approaches were used: two conventional and one proposed EMD-based hybrid approach. Traditional methods for humidity modeling rely heavily on influential parameters related to humidity, like temperature, rainfall, and wind speed. In approach one, data for maximum and minimum temperatures, rainfall, and wind speed have been collected from the Bangladesh Meteorological Department. This would be data that is intact and untainted by the preprocessing phase, hence more suitable for further analysis and interpretation. These parameters are presented as facts because they have a direct influence on the atmospheric conditions that drive humidity. The next approach used by traditional humidity modeling, apart from the temperature, rainfall, and wind speed parameters, is a 95 % significant lag value. Whereas these approaches drive structured views, past research has identified major limitations of such traditional approaches. Traditional methods very often miss out on the intricate patterns of humidity since they carry a high level of nonlinearity and noise in the data [37]. The challenge associated with humidity prediction is the removal of noise from the data [38]. In this research, an EMD-based denoising approach was applied to solve this problem, ultimately providing more accuracy in the predictions [36]. EMD represents advanced signal processing techniques that decompose a signal into intrinsic mode functions [33,39,40], which are different frequency components constituting the original signal. This decomposition helps in isolating and removing noise to reveal the real underlying patterns inherently conveyed by the data [41,42]. Often, noise is manifested in the higher frequency IMFs, while the key trends and patterns of the humidity data are held by the lower frequency IMFs. These were analyzed, and the noise components identified were filtered, resulting in a cleaner and closer representation of the original data. Further denoising data used for predictive modeling using SVM: The SVM, indeed, being a far more sophisticated machine learning model, can very ably handle complicated non-linear correlations by determining an ideal hyperplane, which classifies these data points into distinct categories [[43], [44], [45], [46], [47]]. The cleaner this dataset was, the better it would enable the SVM to capture intrinsic intricate patterns in humidity data, reducing prediction errors and hence improving accuracy. So, the primary objective of this research is the application of EMD as a preprocessing approach for enhancing the prediction accuracy of relative humidity.

## 2 Study area and data

**Fig. 1:**
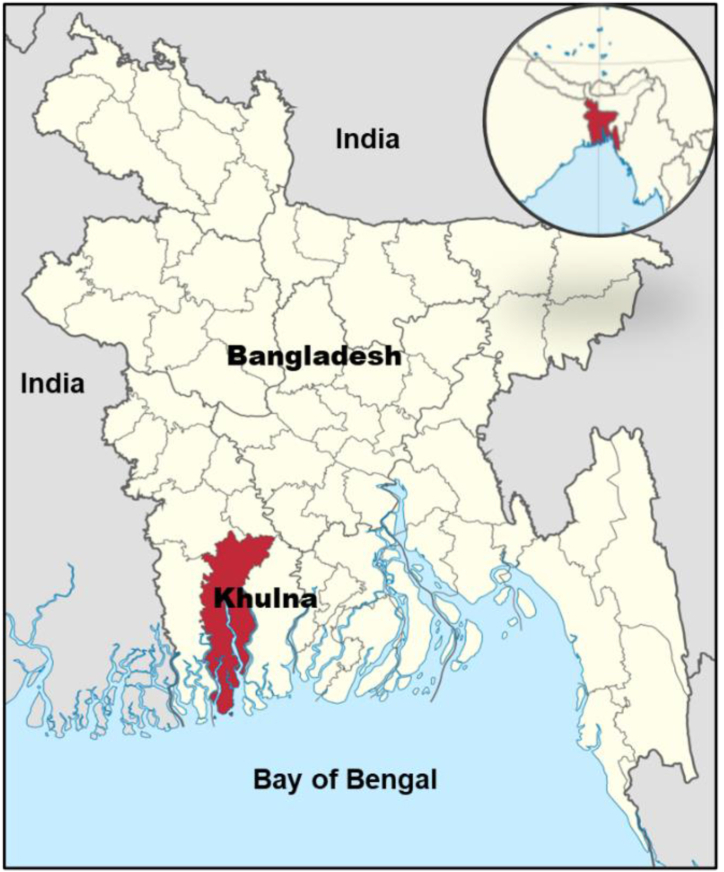
Study area map.

Khulna (latitude 22.8373N, longitude 89.5400E) is located on the northern bank of Rupsha and Vairab in the south-western part of Bangladesh, as shown in Fig. 1. It has more than 9.65 million inhabitants (2024) and a total area of 150.57 square kilometers [48]. Khulna has a tropical wet and dry climate. The city is hot and humid during the summer and pleasantly warm during the winter. Khulna is significantly affected by the monsoon in South Asia [49]. Its annual average rainfall is 1878.4 mm (73.95 in) h about 87 percent falling between May and October [27]. Khulna also receives heavy rain from cyclones that form in the Bay of Bengal. The city has an annual average temperature of 26.3 °C (79.3 °F), with monthly averages ranging from 11.4 °C (52.5 °F) on January mornings to 34.6 °C (94.3 °F) during April afternoons [49]. This city is also congested with high-emitting vehicles and lots of small-scale factories. This city has the least amount of rainfall of any other city in Bangladesh. The city is also facing an alarming rate of air pollution. The construction of buildings, roads, and highways is also a great source of air pollution. Some other factors, such as smoke from industries, badly maintained motor vehicles, coal burning, brick manufacturing, construction works, and municipal waste dumping, have a great impact on air pollution. Air quality depends on some significant parameters. Particulate matter (PM) is one of the most widely studied parameters as it has a strong correlation with health outcomes due to its high concentrations in Khulna city. According to the Ministry of Environment (Ambient Air Quality Project 2017), PM10, PM2.5, CO, SO2, NOX, and O3 are the most commonly used meteorological parameters to analyze air pollution [50]. These meteorological parameters contribute to air pollution as well as accelerating temperature change. Humidity is inversely proportional to temperature [51].Apart from these, there are various factors that primarily affect temperature and humidity. Khulna is a major city in Bangladesh due to the rapid growth of populations, industries, etc. Many.

Factors have caused rapid environmental changes in Khulna. The Padma Bridge Project is alleviating the burden of population, industries, and traffic in Dhaka, the capital of Bangladesh, by facilitating easier communication [52]. It makes environmental changes in this area, and now it's a very concerning issue of Khulna. So, being a growing city, it needs sustainable development. Therefore, this environmental analysis is going to hugely affect saving the environment from rapid changes. Besides, Khulna is also a large area under crops. Due to unusual changes in climate variables, farmers are suffering problems and are demotivated from cultivation. This will affect the economy due to the unusual changes in temperature and rainfall during summer and winter seasons. This makes it a harder situation for the public in general; therefore, the accurate prediction of relative humidity will greatly influence sustainability to aid in the making of decisions to decrease these climate changes.

**Fig. 2:**
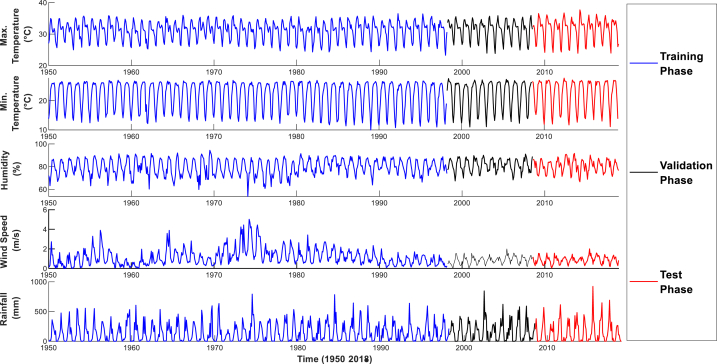
Time series plot of different input-output variables adopted for humidity prediction.

In this study, data regarding meteorological parameters like monthly relative humidity, rainfall, maximum and minimum temperature, wind speed, etc. have been obtained from the Bangladesh Meteorological Department (BMD) from 1950 to 2018 [53]. There are 35 meteorological stations in Bangladesh; Khulna Station is one of them (Meteorological Station ID: 419310-99999). Besides, this organization is a very dependable source in Bangladesh. Different meteorological variables in Khulna are measured at this station. The sampling period has a considerable impact on the final result. A longer period of time or at different times of year may provide deeper insights into humidity trends [54]. That is why a 69-year period, from 1950 to 2018, was used for analysis. The dataset spanning 1950–2018 was divided into three phases: training, validation, and testing, with a 70:15:15 ratio. In the data, 70 % of the series was used for training from January 1950 to April 1998, with the remaining 15 % preserved for validation from May 1998 to August 2008. The model's performance was then evaluated using the last 15 % of the data from September 2008 to December 2018 as the test set after training and validation. The collected variables and different phases shown in Fig. 2. After collecting data, the data was arranged in a time series format for further analysis. The preprocessing phase of the collected data was quite stringent, involving cleaning the data for accuracy and consistency. This involved the identification and rectification of anomalies or errors in the dataset, handling missing values, and checking for integrity in the analysis. The preprocessing steps were therefore very important in preparing data for further analysis, meaning that the results derived from the study had a backbone of robust and reliable information. This fact that meteorological parameters were extracted from BMD, gave a very impressive level of confidence in the quality and reliability of the data used for the study, hence providing appropriate analysis of the climatic conditions under investigation.

## 3 Methodology

**Fig. 3:**
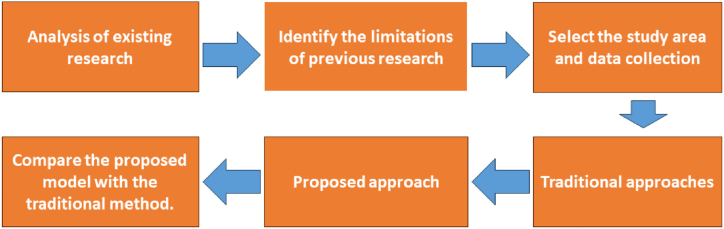
Flowchart of the adopted overall methodology in this study.

The current research methodology is illustrated in Fig. 3. At the beginning, from the literature review, background knowledge has been gained about different traditional techniques in humidity prediction. From the analysis of previous studies, it was found that the main challenge is dealing with noisy data. Previously, humidity prediction took place in such kinds of geological locations where rapid climate change and urbanization occurred. The study focused on the location in Khulna, which is the fastest-growing city in Bangladesh. Due to the environmental challenge, it is very necessary to predict environmental components in this region. The necessary data collection and initial processing are described previously. The study developed three approaches for humidity modelling; two are traditional, and one is the proposed method, which takes the challenge to overcome the limitation of traditional method.

### 3.1 Traditional methods

The two traditional methodologies employed to improve predictive accuracy in humidity patterns. The first one is the conventional methodology, which applies the different climate components considered to affect humidity directly. These include, for instance, maximum temperature, minimum temperature, wind speed, and rainfall, which help to discern the multivariate interaction between multiple climate variables. These are very important components that provide a form of baseline comparison against more advanced techniques for discerning the multivariate interaction between multiple climate components [27].

The second method under the traditional method is the lag-time-based approach. In most conventional methods of prediction, the lag time is used as a feature input, a very familiar strategy that has been comprehensively surveyed and put into practice rather abundantly in prediction problems [55,56]. For example, in studies [56,57], and [58], it used lag time as an input feature. Lags simply refer to the historical observations for a particular variable used as a predictor. Since the given PACF plot illustrated in Fig. 6 shows significant lags 1, it can be inferred that those lags have meaningful correlations with the current value of the humidity time series. This study uses data from the preceding time points, t-1 as features in training a model for estimating the amount of humidity at a given time by the following Eq. (1):

(1) $$y_{t}=f\;\left(y_{t-1}\right)+\in$$

Where, y_t_ is the value of the time series at time, 't', 'f' is the function learned by the machine learning model, y_t-1_ is the lagged values of the series; ' ∈' is an error term that refers to any kind of noise or pure randomness that cannot be predicted by the model.

### 3.2 Proposed hybrid method

The study suggests a hybrid technique for humidity prediction. The last two traditional methodologies have some disadvantages, including the presence of noise in the data. These two strategies apply data directly to the predictive model. The proposed method included preprocessing steps before applying it to the model via Empirical Mode Decomposition (EMD). After breaking down the historical humidity data into multiple frequency components, the Support Vector Machine (SVM) is used to create the predictive model. The EMD is a well-known noise reduction technique used in signal processing. This study aims to determine the applicability of signal processing elements for relative humidity prediction in the Khulna region.

#### 3.2.1 Empirical mode decomposition (EMD)

**Fig. 4:**
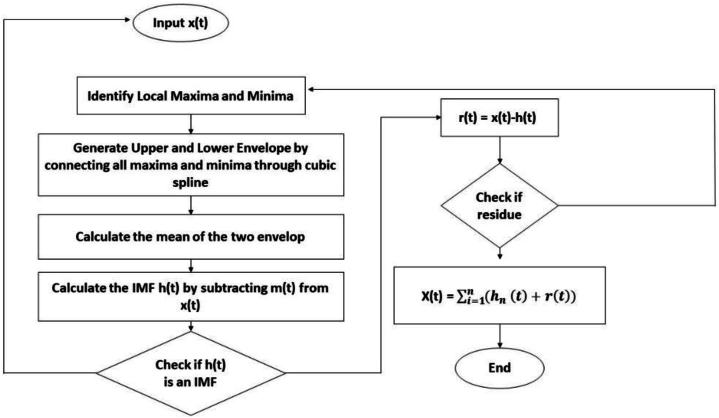
The flow chart of the EMD process.

Empirical Mode Decomposition (EMD) is an algorithm that is used to separate a composite signal into different components that have a physical meaning [35]. EMD is mainly a signal decomposition technique that divides the actual complex signals into different subcomponents known as Intrinsic Mode Functions (IMFs) [59]. This method is very effective for complex data analysis. These physically meaningful components are called the Intrinsic Mode Functions (IMFs). So, EMD decomposes a multi-scale nonlinear, non-stationary signal into a finite number of intrinsic mode functions [60]. It can remove noise from the signal to get the effective signal [36]. The EMD approach consists of several steps that mainly starts with the humidity value. EMD then finds the maximum and minimum nodes in the signal, creates upper and lower envelopes using cubic spline curves, and computes a mean curve by averaging these envelope values. An IMF has one maximum and one minimum between two subsequence zero crossings, and another is that it has a mean value of zero, which are the main basic properties of IMFs. The EMD working process is illustrated in Fig. 4.

In the past, many publications have used the empirical mode decomposition methodology for handling highly nonlinear patterns in different sectors. These studies give examples of the strength of EMD in decomposing any complex signal into easier components that are more understandable to perceive and, hence, more efficiently analyzed and forecasted. These applications further underline the strength of EMD in handling a wide variety of nonlinear and complex data sets and, therefore, bring improved predictive capabilities to most disciplines. Results from such studies have firmly established EMD as one of the most efficient methods for filtering noise and enhancing the reliability and accuracy of models on environmental and operational datasets, thus offering a sound basis for decision- and management-making within most scientific and engineering applications. The mathematical concept behind the EMD process is described below:

**EMD Algorithm:** Decompose a data set x(t) into IMFs h_(n)_(t) and a residuum r(t) such that the signal can be represented as Eq. (2).

(2) $$x\left(t\right)=\sum_{i=1}^{n}\left(h_{n}\left(t\right)+r\left(t\right)\right)$$

Steps

(1) Extract all local maxima and minima of the function x(t).
(2) Step 1 developed by using the cubic spline interpolation the lower envelope e_l_(t) defined as the minima and the upper envelope e_u_(t) defined as the maxima.
(3) Calculate the mean function of the maxima and the minima m(t)= (e _(u)_ (t) + e_(i)_ (t))/2.
(4) Let, d(t) = x(t) – m(t), if d(t) is the zero mean function then the iteration stops. Now this d(t) is called the first IMF i,e h(t) = d(t)
(5) If d(t) is not the zero mean function then the steps 1–4 all repeated until there is an IMF.

After breaking down the humidity data into distinct components, the IMFs and residue are used for model training with the Support Vector Machine (SVM), a well-known machine learning approach that has previously been employed in a variety of applications.

#### 3.2.2 Support vector machine (SVM)

Support Vector Machine (SVM) is a sophisticated machine learning algorithm that may be applied to linear or nonlinear classification, regression, and outlier detection [61]. SVMs have a wide range of applications, including text classification, picture classification, spam detection, handwriting identification, gene expression analysis, face detection, and anomaly detection. SVMs are useful and efficient in a variety of applications because they can handle high-dimensional data and nonlinear relationships [62].

SVM identifies the extreme points/vectors that help form the hyperplane [63]. These extreme cases are referred to as support vectors, and the algorithm is called Support Vector Machine. Consider the following diagram, which depicts two distinct groups classified using a decision boundary or hyperplane: The research by Refs. [64,65] encouraged the study to employ the Support Vector Machine, SVM model in handling the extremely nonlinear data of rainfall and temperature. Additionally, the SVM is especially effective in capturing the complex nonlinear patterns that are usually typical under the climate variables [66]. It provides high accuracy and is pretty robust against overfitting and hence ideal for both small and medium-sized datasets. Also, the fact that the model can be applied in both regression and classification makes it more convenient in many weather prediction problems [67]. In SVM, the parameters of regularization act as an important control that can be used to obtain a fit that has small empirical errors on the data used for training and still get a good generalization on new data [68]. The regularization parameter, C is a parameter, which trades between the obtained error on the training data and the separability among the classes. A small one implies that one wants to have a smooth decision surface, while a large one allows the model to select more samples as support vectors by leaving more freedom in the model. A small C gives a smooth decision surface, but increases chances of misclassifying even more points [69]. This is high regularization. The high value of C will force to classroom all the examples of the training set correctly; this will lead to overfitting, especially if there is noised. SVM uses kernel functions to efficiently deal with complex transformations of the data and uses structural risk minimization principle so that it generalizes well on new data. In this work, three kernel functions are considered: the linear kernel function in Eq. (3), the Gaussian RBF kernel in Eq. (4), and the polynomial kernel in Eq. (5).

(3) $$\text{Linear}:K\;\left(\omega ,b\right)=\omega^{T}\;x+b$$

(4) $$\text{Polynomial}:K\left(\omega ,x\right)={\left(\gamma \omega^{T}x+b\right)}^{N}$$

(5) GaussianRBF:K(ω,x)=exp(−γǁxi−xjǁ)n

This study incorporated the use of the Particle Swarm Optimizer (PSO) in the optimization of the hypermeters [36]. Swarms needed to be initialized with the aim of finding the best fitted kernel and regularization parameters. This is to be done through each particle in the swarm, which corresponds to a potential solution, including the selection of the kernel and the C value. Also, the search space has to be defined. In this way, for modeling a linear, polynomial, and Gaussian RBF, their search space is defined through the range of regularization parameters. The objective function to minimize might be the error rate on a validation set or cross-validation accuracy. This function really evaluates how well the SVM with the given parameters is performing. Randomly initialize the positions of the particles within the defined search space. Thereafter, an SVM is trained using these parameters given by each particle, and the value of the objective function is checked. The updated velocity and position of each particle are given by the following Eqs. (6), (7) of PSO:

(6) $$v_{i}\left(t+1\right)=\omega v_{i}\left(t\right)+c1r1\left(p_{i}-x_{i}\right)+c2r2\left(g-x_{i}\right)v_{i}\left(t+1\right)=\omega v_{i}\left(t\right)+c1r1\left(p_{i}-x_{i}\right)+c2r2\left(g-x_{i}\right)$$

(7) $$x_{i}\left(t+1\right)=x_{i}\left(t\right)+v_{i}\left(t+1\right)x_{i}\left(t+1\right)=x_{i}\left(t\right)+v_{i}\left(t+1\right)$$

**Fig. 5:**
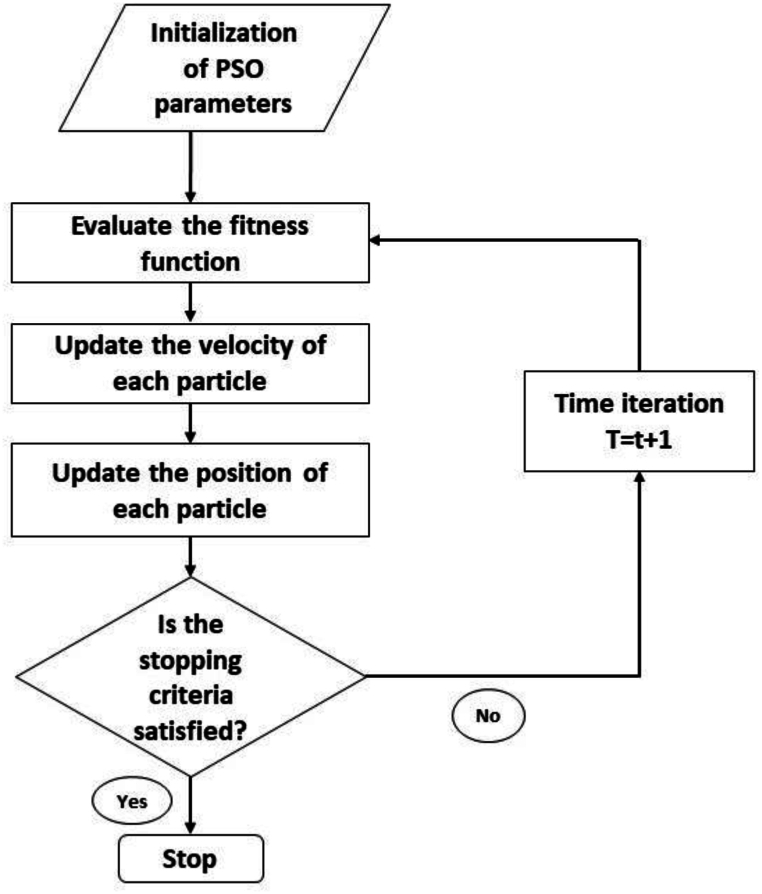
Working process of the PSO algorithm.

Here, *vi*(*t*),vi(t) is the velocity of particle *i*i at time *t*t. *xi*(*t*), xi(t) is the position of particle *i*i at time *t*t. *pi*, pi is the best-known position of particle *i*i. *g*g is the global best position found by any particle. ***ω***ω is the inertia weight. *c*1, c1 and *c*2, c2 are cognitive and social coefficients. *r*1, r1 and *r*2, r2 are random numbers between 0 and 1. After that repeat the evaluation and update steps for a fixed number of iterations or until the change in the objective function is below a certain threshold. Fig. 5 illustrates the flow chart of PSO. The model performances were evaluated by MSE, RMSE, MAE, MAPE and R2.

**Fig. 6:**
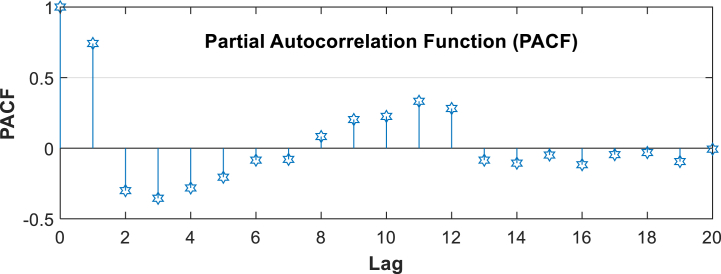
PACF plot of Relative Humidity.

MSE (Mean Squared Error) is the measurement of average square of errors between the actual and theoretical values in regression model. MSE is a commonly used regression model that ensures the accuracy of prediction, decision making and optimization of the model given in Eq. (8).

(8) MSE=∑i=1i=n(yi−y)2

n = number of possible data sets, yi = actual value of yth sample, y = estimated/Theoretical value of yth sample. RMSE (Root Mean Squared Error) is also a method that is used to determine the performance and accuracy of regression model, as shown in Eq. (9). It is more sensitive in compare to MSE. It can analyze and compare many variables. Due to squared value of errors the sensitivity and accuracy is high in that mathematical method.

(9) RMSE=MSE=∑i=1i=n(yi−y)2

n = number of possible data sets, yi = actual value of yth sample, y = estimated/Theoretical value of yth sample. R^2^ which is known as the coefficient of determination, is a statistical measure used in regression analysis to assess how well the independent variables explain the variability of the dependent variable in a regression model, as shown in Eq. (10). R^2^ indicates how accurate the model fits the observed data & it can also provide insights into the model's predictive power. A higher R^2^ suggests that the model may perform better in predicting future data.

(10) $$R^{2}=1-\;\frac{SStotal}{SSresidual}$$

SStotal = Total Sum of Squares of the deviation of each observed value from the mean value. SSresidual = Sum of Squared Errors. MAE (Mean Absolute Error) is the average of the absolute difference between the actual value and the predicted theoretical value of any given data, as shown in Eq. (11). MAE performs a vital role in the comparing and improving the predicted model in various engineering calculations. It performs high accuracy in lower amount of data and vice-versa.

(11) $$MAE=\frac{1}{n}\sum_{i=1}^{i=n}\;\left|yi-xi\right|$$

yi = Actual value, xi = predicted value, n = number of possible data sets.

MAPE (Mean Absolute Percentage Error) is the mean of all absolute percentage error between the actual and predicted value, as shown in Eq. (12). It is similar to MAE, but result expressed in percentage. It is mainly scale invariant and emphasizes on relative errors rather than absolute errors.

(12) $$\text{MAPE}=\frac{1}{n}\sum_{i=1}^{i=n}\;\left|\frac{yi-xi}{yi}\right|\times 100\%$$

yi = Actual value, xi = predicted value, n = number of possible data sets.

## 4 Result and discussion

The primary purpose of the work is to evaluate the application of EMD with the SVM algorithm in predicting air humidity in Khulna, Bangladesh. This site is facing a warming climate due to rapid urbanization. Fig. 2 illustrates the complex and non-linear humidity pattern in this area. The prediction of relative humidity helps to know the humidity pattern and use this information for sustainable development in this area. The combined impacts of rapid urbanization, global climate change, and deforestation require the core of this study to be on humidity prediction in this region. To predict the meteorological data, the model was designed by the Support Vector Machine. The collected data were arranged in a time series format from 1950 to 2018. While modelling the humidity, the initial 70 % (January 1950–April 1998) of the data is set as the training data for the model. The model takes 15 % (May 1998–August 2008) of the data for the validation phase. The model was trained on the training data, and later, during the validation phase, it was checked by finding the best hypermeters using Particle Swarm Optimization (PSO). At the end of training and validation, the model was run to predict test data, whereby the model used the last 15 % (September 2008–December 2018) of the data as test data. For this, the performance of the SVM algorithm is decided by the model hyperparameters, like kernel functions and regularization parameters. In the current study, different kernel functions have been used, including linear, Gaussian RBF, and polynomial kernels. The range of regularization parameters has also been added to the model to optimize these parameters. Further, the particle swarm optimization algorithm has been employed for finding optimal hyperparameters, or the best-fitted kernel and regularization parameter. The result of the proposed EMD-based hybrid ML model was compared with two traditional approaches, such as the use of different relevant meteorological parameters that influence the humidity level in Khulna and the lag-time-based approach, which correlates the previous time steps of humidity data.

The first traditional approach consists of various input features such as maximum temperature (M.T.), minimum temperature (Min.T.), wind speed (W), and rainfall (R). These features are crucial for accurately predicting humidity because of their direct correlation with humidity. The research [27] illustrates a significant correlation between these variables in the Khulna area. These influential meteorological parameters, especially rainfall and temperature, are also rapidly changing in this region, as suggested by research [70]. That's why it is wise to use this variable to predict humidity in Khulna. The correlation matrix shown in Table 1 illustrates that all the variables are positively correlated with the humidity. The minimum temperature and rainfall have a very significant correlation with humidity. The different input combinations were selected by the correlation matrix shown in Table 1, Table 2 provides the performance of different input combinations in terms of MSE, RMSE, MAE, MAPE, and R2 values. Additionally, it specifies the best kernel and the optimal C and gamma values determined by PSO for each model configuration.

**Table 1:** Correlation matrix of different input and output variables.

	Max. temperature	Min. temperature	Wind speed	Total Rainfall	Humidity
Max. temperature	1				
Min. temperature	0.83	1			
Wind speed	0.41	0.41	1		
Total Rainfall	0.32	0.65	0.30	1	
Humidity	0.12	0.56	0.09	0.68	1

**Table 2:** Performance analysis of three different methods.

Model	Input	MSE	RMSE	MAE	MAPE	R2	Best Kernel	Best C	Best Gamma
H1	M.T, Min.T, W, R, H	9.39	3.06	2.15	2.67 %	0.76	Gaussian	1	1
H2	M.T, Min.T, R, H	9.61	3.1	2.21	2.74 %	0.77	Gaussian	1	1
H3	Min.T, R, H	19.2	4.38	3.5	4.45 %	0.52	Gaussian	0.1	1
H4	R	17.26	4.15	3.42	4.32 %	0.59	Gaussian	0.1	1
H5	Min. T	27.35	5.23	4.05	5.19 %	0.56	Gaussian	0.1	1
H6	T-1	16.59	4.07	3.32	4.17 %	0.56	Linear	0.1	1
EMD	EMD	0.21	0.46	0.38	0.46 %	0.92	Linear	10	0.01

The configuration combining max. temperature, minimum temperature, wind speed, and rainfall (M.T, Min.T, W, R–H1) shows strong performance metrics with an MSE of 9.39, RMSE of 3.06, MAE of 2.15, and MAPE of 2.67 %. It achieves a high R2 value of 0.76, indicating a robust predictive capability. The optimal SVM parameters for this configuration, identified by PSO, are a Gaussian kernel with C = 1 and Gamma = 1. When the input is reduced to max. temperature, minimum temperature, and rainfall (M.T., Min.T., R–H2), the model still performs well, with an MSE of 9.61 and slightly higher RMSE and MAE values. This configuration also uses a Gaussian kernel optimized with the same C and Gamma values. The R2 value is 0.77, demonstrating reliable performance. For the configuration using only minimum temperature and rainfall (Min.T, R–H3), the performance drops, as indicated by an increased MSE of 19.2 and RMSE of 4.38. The R2 value is 0.52. This decline in performance suggests that the absence of maximum. temperature and wind speed data reduce the model's accuracy. The model with only rainfall (R–H4) as input performs slightly better than the previous configuration but still lags significantly behind the top performers. It has an MSE of 17.26, an RMSE of 4.15, a MAE of 3.42, a MAPE of 4.32 %, and an R2 of 0.59. PSO identifies the Gaussian kernel with C = 0.1 and Gamma = 1 as optimal parameter for this configuration. Using only the minimum temperature (Min. T-H5) results in the poorest performance metrics, with an MSE of 27.35 and an RMSE of 5.23. This configuration's R2 value is 0.56, showing it is not sufficient for accurate predictions. PSO optimization again favors a Gaussian kernel with C = 0.1 and Gamma = 1.

In the second conventional technique of humidity prediction, the lag time of humidity data is employed as a feature input, a highly familiar strategy that has been studied and widely used in prediction problems [55,56]. For example, in studies [56,57], and [58], lag time was used as an input feature. Lags simply refer to the historical observations for a particular variable used as a predictor. Since the given PACF plot illustrated in Fig. 6 shows significant lag1, it can be inferred that those lags have meaningful correlations with the current value of the humidity time series. In the instance of the humidity prediction, it is observed that its lag 1 is significant, meaning that the previous time period, for instance, last month's humidity, is very influential on the one that occurred in the current time period. The configuration using the previous month's humidity (T-1-H6) and considering the PACF plot shows moderate performance with an MSE of 16.59, RMSE of 4.07, MAE of 3.32, and MAPE of 4.17 %. It achieves an R2 value of 0.56 using a linear kernel optimized with C = 0.1 and Gamma = 1.

The proposed approach based on the EMD can improve prediction accuracy more than the traditional approaches. EMD is a technique that can split the data into different frequency components. SVM can capture the intricate humidity pattern using these different frequency components. In traditional approaches, SVM tried to make relationships directly between different features, whereas in the proposed method, the EMD can provide the core humidity patterns of different frequencies to the SVM model. Thus, the SVM captures the humidity pattern more precisely than the conventional method. The proposed approach shows a standout performer is the Empirical Mode Decomposition (EMD) input configuration, which achieves exceptional accuracy with an MSE of 0.21, RMSE of 0.46, MAE of 0.38, and MAPE of 0.46 %. It boasts an R2 value of 0.92. For this model, PSO identifies a linear kernel with C = 10 and Gamma = 0.01 as optimal parameters.

Compared with the traditional models H1 to H6, the EMD model contributes to a great improvement in predictive accuracy, as shown by the substantially increased R2 values. Therein, H1 takes the predictor variables as maximum temperature, minimum temperature, wind speed, rainfall, and humidity, with its R2 value standing at 0.76, while it will boost to 0.92 if replaced by the EMD model, rising by 21.05 %. The H2 model, without wind speed, exceeded the R2 value only slightly, at 0.77, while that for the EMD model increased by 19.48 %. More dramatic is the increase seen in the H3 model, which used only minimum temperature, rainfall, and humidity: its R2 jumped from 0.52 to 0.92, an incredible 76.92 % rise. In the case of the H4 model, dependent solely on rainfall, the EMD model raised the R2 from 0.59 to 0.92, a 55.93 % improvement. This means that, for the H5 model with only a minimum temperature, the R2 jumps from 0.56 to 0.92, an increase of 64.29 %. Similarly, for the lagged H6 model in one of the temperature variables, the R2 increased from 0.56 to 0.92 again, for an increase of 64.29 %.

The improvement in accuracy compared to the traditional method illustrates that the EMD can help SVM capture patterns accurately. One of the primary challenges is handling environmental components such as humidity because of the existence of noise [71]. Due to rapid environmental events, instrumental errors, and different unusual conditions, it is not possible to measure the meteorological components; it makes a nose in the data [71]. It is very difficult to find the pattern. The traditional approaches directly used this noisy data, which made the error in predicting humidity higher [72], but the EMD can break the noisy data into different higher and lower frequencies called IMFs. More specifically, EMD decomposes data into IMFs, separating the higher-frequency components from the lower-frequency ones and describing the underlying trends and slower variations in the data [73]. The different IMFs are shown in Fig. 8. It thus allows a deeper view into the characteristic features of the signal. That means the decomposition process brings out different phases of the data, and hence the detection of the intricate features and patterns is easy, although hidden in noise [74]. Besides the intrinsic mode functions, a very vital component of EMD is the residue [36]. The residue shows the general trend or may be considered a component having the slowest variability of any original signal, thus capturing long-term behaviour remaining after all intrinsic mode functions have been filtered out [75]. Isolation of this residue may help in focusing on the trend underlying the data and turn out to give a clear view of the inherent features of the data [76]. Applied to humidity data, this may improve the detection of subtle variations and trends and hence give a clearer understanding of the underlying phenomena. The SVM can be analyzed by taking more deep information through this process, and from the analysis, it was shown that the EMD-SVM gives the lowest error performance with a highest R2 value.

**Fig. 7:**
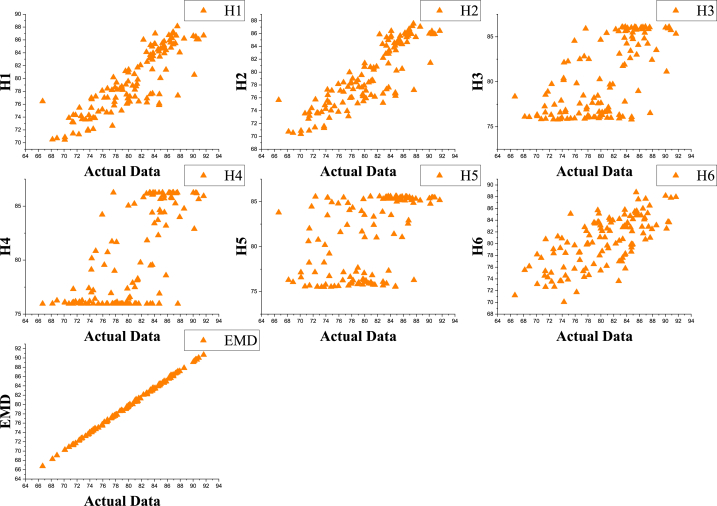
Correlation of the actual and predicted values of different models.

This significant increase thus shows how the EMD model offers enhanced ability to capture complicated patterns within the data and therefore gives better accuracy and reliability in the forecasting process. Moreover, the use of an optimal parameter-based linear kernel in the EMD model underscores its potency and efficiency in handling meteorological data, rendering it effective support in conducting climate analysis and prediction in Bangladesh. Fig. 7 illustrates the scatter plot of the actual and predicted values of different models, and Fig. 9 is a pair, that is, a scatterplot matrix showing the relationships between variables. Plots Each subplot is a scatter plot of two variables. There are variables' names listed along the diagonal. The plot enables one to quickly scan for possible correlations or patterns between variables. The resulting pair plot is a nice way of visualizing multiple variables for relationships and distribution and thus will illuminate significant patterns, correlations, or issues, such as outliers [77]. The study has some limitations, such as the traditional method, which used different influential parameters related to humidity in Khulna and used limited input variables. Researchers can analyze further by considering additional variables. There is an updated version of EMD called Ensemble Empirical Mode Decomposition (EEMD). Researchers can further analyze it by taking the EEMD or other noise reduction approaches. This study used only the particle swarm optimization algorithm for finding optimal SVM hybrids. Researchers can further use different optimization algorithms. The use of different optimization techniques can enhance the accuracy of the proposed model. To develop the SVM model, a fixed number of regularization parameters was used. The use of a large range of regularization parameters may help to improve the accuracy. During humidity prediction in Khulna, the study suggests taking into account these limitations.

**Fig. 8:**
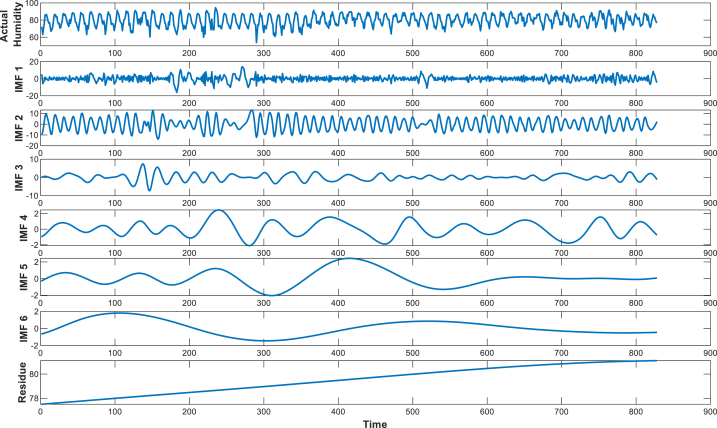
Decomposed signal into six IMFs and residual.

**Fig. 9:**
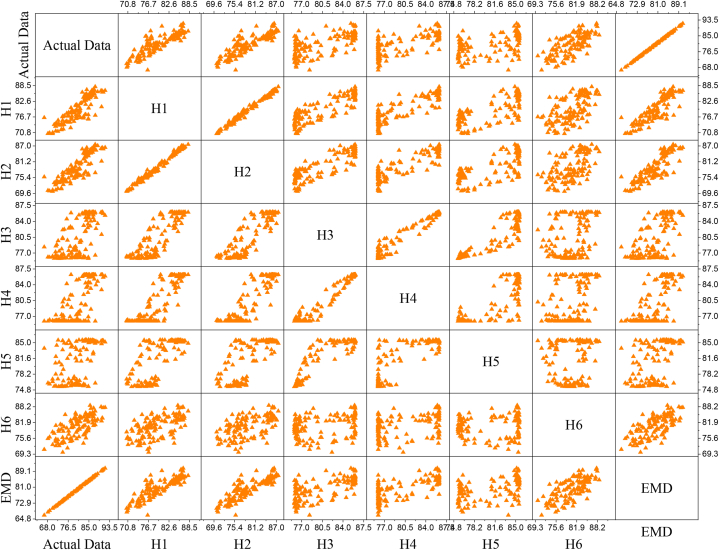
A Scatterplot matrix of different variables.

## 5 Conclusion

This current research focused on noise removal by analyzing the applicability of the empirical mode decomposition method combined with the support vector machine algorithm in air humidity prediction in Khulna, Bangladesh. This city has been under the scrutiny of fast urbanization and climatic change. The results obtained from this study indicate that the proposed EMD-based SVM model significantly enhances predictive accuracy compared to traditional methods. The developed EMD-SVM model gave very outstanding performance metrics, which include an MSE of 0.21, an RMSE of 0.46, a MAE of 0.38, a MAPE of 0.46 %, and an R2 value of 0.92. The performances show a major improvement over the conventional models, including the configuration that combines maximum temperature, minimum temperature, wind speed, and rainfall with an R2 of 0.76. The dramatic increases in the R2 values across all configurations show the improved capability of the EMD model in capturing complex humidity patterns. The major advantage of the EMD approach is its ability to decompose data into different frequency components so that the SVM can capture the intricate humidity patterns more precisely. Traditional methods struggled with noisy data and, hence, higher prediction errors. Contrarily, EMD has the knack of insulating a few phases of the dataset, bringing out the underlying trend, and helping the SVM reduce error rates and increase the accuracy of the model. Despite this success, some limitations of this study are the few numbers of input variables used by the traditional approach; there may be other variables that can still be added in future studies to increase predictive accuracy. Further, this research was done only with PSO as an optimization tool to find appropriate parameters for SVM. Searching for other optimization methods may lead to better results. Moreover, it was searching in the fixed range for regularization parameters; it may lead to the discovery of better results by extending this range too. The result shows that the EMD-based SVM model is a very strong and reliable tool for predicting humidity in Khulna, with huge implications for sustainable development and climate adaptation strategies. Future studies should include the inclusion of other noise reduction techniques, like EEMD, to improve the model. It will enhance the model's accuracy and reliability in humidity prediction, leading to effective climate analysis and forecasting.

## Research support

The research received no external financial or non-financial support.

## Relationships

There are no additional relationships to disclose.

## Patents and intellectual property

There are no patents to disclose.

## Other activities

There are no additional activities to disclose.

## Data availability statement

Data will be made available on request.

## CRediT authorship contribution statement

**Shuvendu Pal Shuvo:** Writing – review & editing, Writing – original draft, Validation, Software, Methodology, Investigation, Formal analysis, Data curation, Conceptualization. **Joarder MdAshikuzzaman:** Writing – review & editing, Writing – original draft, Formal analysis, Conceptualization. **Shirshendu Pal Shibazee:** Writing – review & editing, Writing – original draft, Methodology, Investigation, Formal analysis, Conceptualization. **Goutam Paul:** Writing – review & editing, Writing – original draft, Formal analysis, Conceptualization. **Pritam Banerjee:** Writing – review & editing, Writing – original draft, Formal analysis, Conceptualization. **Kazi Mashfiq Fahmid:** Writing – review & editing, Writing – original draft, Formal analysis, Conceptualization. **Ashiqur Rahman:** Writing – review & editing, Writing – original draft, Formal analysis, Conceptualization.

## Declaration of competing interest

The authors declare that they have no known competing financial interests or personal relationships that could have appeared to influence the work reported in this paper.
